# Lensless Computational Imaging Technology Using Deep Convolutional Network

**DOI:** 10.3390/s20092661

**Published:** 2020-05-06

**Authors:** Peidong Chen, Xiuqin Su, Muyuan Liu, Wenhua Zhu

**Affiliations:** 1 CAS Key Laboratory of Space Precision Measurement, Xi’an Institute of Optics and Precision Mechanics, Chinese Academy of Sciences, Xi’an 710119, China; chenpeidong18@mails.ucas.ac.cn (P.C.); liumuyuan18@mails.ucas.ac.cn (M.L.); zhuwenhua15@mails.ucas.ac.cn (W.Z.); 2 University of Chinese Academy of Sciences, Beijing 100049, China

**Keywords:** lensless, lens-free, computational imaging, deep learning, FCN (Fully Convolutional Networks), U-Net, Dense-U-Net, image reconstruction

## Abstract

Within the framework of Internet of Things or when constrained in limited space, lensless imaging technology provides effective imaging solutions with low cost and reduced size prototypes. In this paper, we proposed a method combining deep learning with lensless coded mask imaging technology. After replacing lenses with the coded mask and using the inverse matrix optimization method to reconstruct the original scene images, we applied FCN-8s, U-Net, and our modified version of U-Net, which is called Dense-U-Net, for post-processing of reconstructed images. The proposed approach showed supreme performance compared to the classical method, where a deep convolutional network leads to critical improvements of the quality of reconstruction.

## 1. Introduction

As the manufacture of a lens strongly depends on the complex mechanical structure and polishing, coupled with expensive infrared and ultraviolet lenses, the cost of traditional optical lens systems is difficult to reduce. Given the rapid development of the Internet of Things and the increasing importance of image and video data, imaging equipment is required to meet the demands in various applications, such as wearable imaging devices, space-constrained scenes, ultra-thin smartphones, and biological imaging, where thin, lightweight, low energy consumption and affordable cost would be considered as the key qualities of imaging equipment. However, current mobile imaging modules are still far from meeting these demands due to the restrictions of the lens.

One of the current solutions to deal with the mentioned problems is the adaptation of lensless imaging technology. At present, lensless imaging technology attempts to replace the physical lens with optical devices such as a spatial light modulator, diffraction optical element, coded mask to achieve the function of light transmission and scene focusing. Compared with other devices, the coded mask is an ideal solution to simplify manufacturing process, low cost, and easier integration with image sensors. Therefore, replacing lenses with a coded mask in imaging equipment can achieve a thinner and more compact structure.

There are many pieces of research on lensless computational imaging technology, and many of them have achieved great results. Replacing lenses with a coded mask has been mentioned in many articles [1,2,3,4,5]. Deweert M.J. et al. presented a new class of coded apertures, called separable Doubly-Toeplitz masks, which are efficiently decodable even for very large images [1]. Asif S. et al. did a similar job and made a prototype of an ultra-thin lensless camera with detachable masks [2,3]. Zheng Y. et al. presented simulation results on image and depth reconstruction for a variety of 3D test scenes. They adopted the method of encoding aperture imaging and alternating a gradient descent algorithm that jointly estimates a continuous depth map and light distribution of the unknown scene from its lensless measurements [4]. Cielak M.J. et al. reviewed the application of the coded aperture imaging system in X-ray and gamma-ray imaging for radiation detection and highlighted potential improvements for coded-aperture based neutron source localization [5].Some other researchers directly used deep learning technology to reconstruct the image captured by a lensless or scattering imaging system [6,7,8,9,10]. Laura Waller et al. proposed an efficient lensless reconstruction method by interweaving traditional models with deep learning methods [6]. Ayan Sinha et al. and Yuan X et al. proved deep neural networks can be trained to solve inverse problems in computational imaging [7,9]. Yang Y. et al. provided a new solution for lensless imaging through scattering media using transfer learning in DNNs (Deep Neural Networks) [8]. Guohai Situ et al. proposed a hybrid neural network for computational imaging through such thick scattering media [10]. Some researchers have also applied compressed sensing to lensless imaging technology [11,12]. Gill P. et al. introduced the working principle of a lensless smart sensor, and looked forward to its application prospect in the Internet of Things scene [13]. Xiaopeng S et al. developed a method to realize color imaging through scattering media [14]. Ganghun K. et al. used only a sensor for imaging [15].

This technique aims to develop a prototype of ultra-thin computational imaging camera, which replaces lenses with a coded mask, and puts the coded mask before the sensor directly. The coded mask modulates and encodes light information from the scene, then uses algorithm decoding and reconstructing images. This kind of imaging structure can significantly reduce the thickness and volume of the imaging system and can be applied to the Internet of Things and space-constrained scenes according to the requirement of thin, lightweight, low energy consumption, and low cost.

However, the image quality produced by lensless devices is still far below the standard achieved by lens imaging systems. To improve the quality of the lensless image, the image generated by the inverse matrix optimization method [16,17] is considered as a degraded image with the limited performance of the inverse matrix optimization method to solve the ill-conditioned problem. Deep learning technology has many effective applications reconstructing degraded images, so we can apply deep convolutional networks to improve the quality of our reconstructed images.

The main purpose of this article is to combine deep learning methods with lensless imaging techniques to generate a new algorithm that improves the image quality produced by lensless imaging systems. After careful consideration, we applied FCN-8s [18], U-Net [19], and our modified version of U-Net, which we refer to as Dense-U-Net, to train and post-process images that are generated by the lensless imaging system. Dense-U-Net is an extension of U-Net, and it combines the characteristics of U-Net and DenseNet [20]. Experiments illustrate that our method can greatly improve the image quality of lensless imaging technology.

This paper contributes by:

(1) Images reconstructed by previous work are considered as images of degradation due to the limited performance of the inverse matrix optimization method to solve the ill-conditioned problem, and using the end-to-end deep convolutional neural network to train and post-process degraded images. Such a process ultimately improves the visual effect of imaging and image quality evaluation parameters.
(2) A new network was proposed, called “Dense-U-Net”, by combining U-Net with a DenseNet network. The network consisting of a denser connection of layers in the same level allows more contextual information of pixel position to be disseminated and produces a final image with more details than the image trained and post-processed by original FCN-8s and U-Net. This network further improves the visual effect of reconstructed images.

This paper is organized as follow: In Section 2, we systematically outline our imaging theory, including the optimization methods used to calibrate the system transfer matrix and the structure of our proposed deep convolutional network. Then, in Section 3, we show the experimental results by comparing the output images processed by our method with the images produced in the previous work. In Section 4, we discuss the effects of different processing methods and networks, supported by our analysis. Finally, we will summarize the research and demonstrate our prospect of future works.

## 2. Imaging Theory

**Figure 1:**
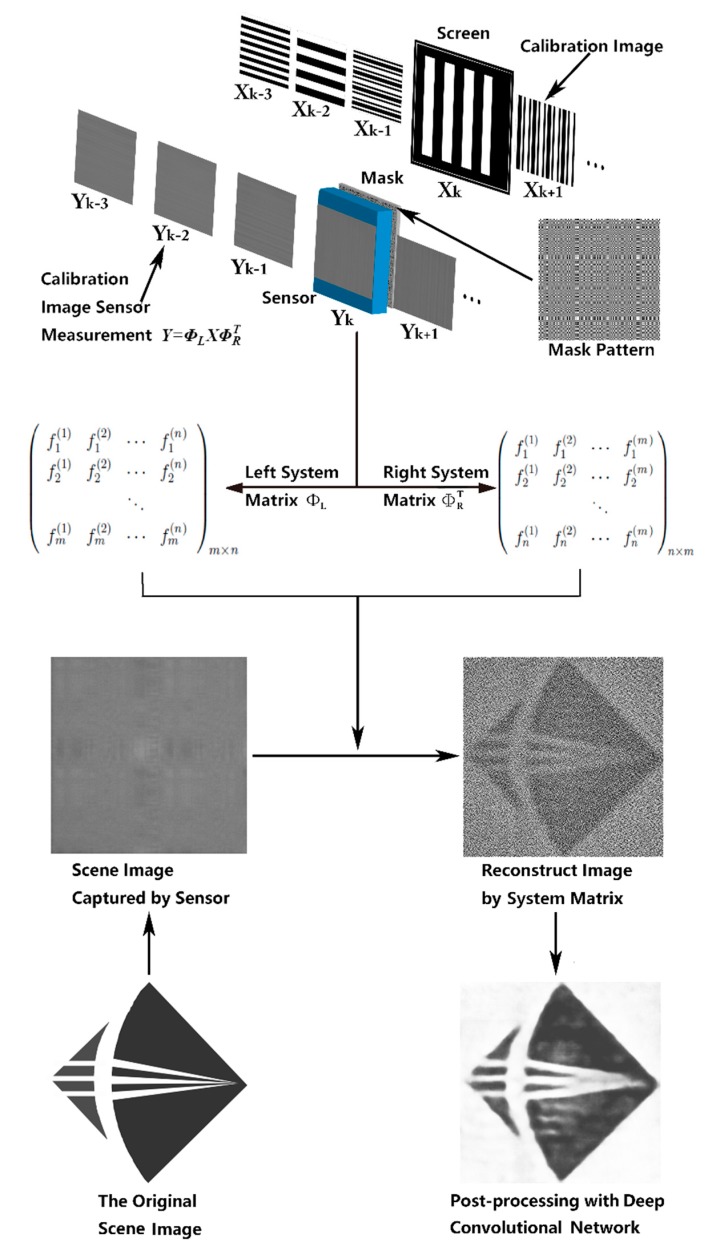
The overall process of image reconstruction and post-processing using a deep convolutional network. As is shown in the figure above, firstly, we used the calibration images measured by the sensor to calculate the left and right system transfer matrix iteratively, and then reconstruct the dataset images and the object images. Next, the dataset images were sent into the deep convolutional network for training, then the trained network was used to post-process the object image, then output the resulting image.

The first step in our lensless image reconstruction method is to reconstruct the measured image through the inverse matrix optimization method, and regard the reconstructed images by the scheme that is proposed in [16] as degraded images. Then, FCN-8s, U-Net or Dense-U-Net are used for training and post-processing the degraded images. We embedded the two steps into a single program, connected by process communication. Figure 1 shows the path of image reconstruction, which consists of calibrating the system transfer matrix with the inverse matrix optimization method and post-process with a deep neural network.

### 2.1. Using Separable Coded Mask to Produce Recognizable Images

The coded mask modulates and encodes light information from a scene, and then we can use an algorithm to decode and reconstruct the measured images. We use Equation (1) to describe the scene-to-sensor mapping, the n-to-n mapping between the scene and the reconstructed image causes solving Φ to be an ill-conditioned problem:

(1) y=Φx+E

where $\Phi \in R^{m\times m\times n\times n}$ is the system transfer matrix, $x\in R^{n\times n}$ is the scene image, $y\in R^{m\times m}$ is the image measured by the sensor, and E represents the sensor noise and model loss. We use the separable coded mask that is proposed in [16] to describe the scene-to-sensor mapping,

(2) $$Y=\Phi_{L}X\;\Phi_{R}^{T}+E$$

where X is the N × N matrix representing the scene, and the total pixels of the scene image is N × N; Y is the M × M matrix representing the sensor measurement, and the total pixels of the sensor measurement image is M × M; $\Phi_{L}$ and $\Phi_{R}$ are left and right system transfer matrices; E is the noise item. After iteratively obtaining the left and right system transfer matrices of the above equation, we can obtain the scene image X from the sensor measurement image Y.

We use Hadamard matrices to generate calibration images, which is composed of “−1” and “1”, and “−1” can be replaced with “0”, and it is a 1 to 1 ratio between the two numbers. The pattern for each calibration image is the product of a column of the Hadamard matrix and the transpose of a vector full of “1”. As for the pattern of the coded mask, we use the 9-order m-sequence algorithm [21] to construct the separable coded mask.

### 2.2. Using Deep Convolutional Network for Post-Processing Degraded Images

Since FCN was proposed in 2015, many researchers have introduced a fully convolutional network structure into the field of feature extraction [22] and image reconstruction and achieved many excellent results.

FCN changed the last several fully connected layers of the traditional CNNs (Convolutional Neural Networks) to a convolutional layer and then used up-sampling operations to output a two-dimensional matrix output (image), while CNNs’ output is one-dimensional information (label), FCN is a series of end-to-end networks (image in, image out).

U-Net was also proposed in 2015. It is a derivative network of FCN. U-Net is also an end-to-end network. The overall structure of the network is similar to the majuscule ‘U’, so it is called U-Net. It consists of the following two parts:

The first part is the feature extraction operations. After each pooling layer operation, the layer scale will be updated. There are a total of 5 scales including the original image’s scale.

The second part is the up-sampling operations. Each up-sampling step is fused in proportion to the number of channels corresponding to the feature extraction part, but it should be clipped before fusion. The fusion here is also a layer concatenation.

DenseNet was proposed in 2017. It connects each layer to each other in a feedback fashion. Whereas traditional convolutional networks, which consist of L layers, have L connections between each layer and the next layer, DenseNet has L(L + 1)/2 direct connections. For each layer, the feature graph of all previous layers is used as input, and its own feature graph is used as input for all subsequent layers. Inspired by the dense links in DenseNet enhancing information dissemination between feature layers, we integrated the U-Net and DenseNet so that our network is both an end-to-end network and a network retaining dense connections for better detail restoration. Therefore, our network is called Dense-U-Net.

We used 18,000 dataset images to train our neural networks, then post-process our object images. These images are captured by a lensless device and reconstructed by previous work. The scene images are from the mini-ImageNet and SelfExSR dataset. The size of all input and output images is 512 × 512 pixels, and all networks have been trained for 6 epochs. MSE (Mean Squared Error) is used as the loss function for training:

(3) $$Loss=\frac{1}{N\times N}{\sum_{0\leq i\leq N}\sum_{0\leq j\leq N}\left({\hat{X}}_{i,j}-X_{i,j}\right)}^{2}$$

where *N* = 512 in our network, $\hat{X}$ represents the output image after each epoch of training, and *X* represents the scene image.

**Figure 2:**
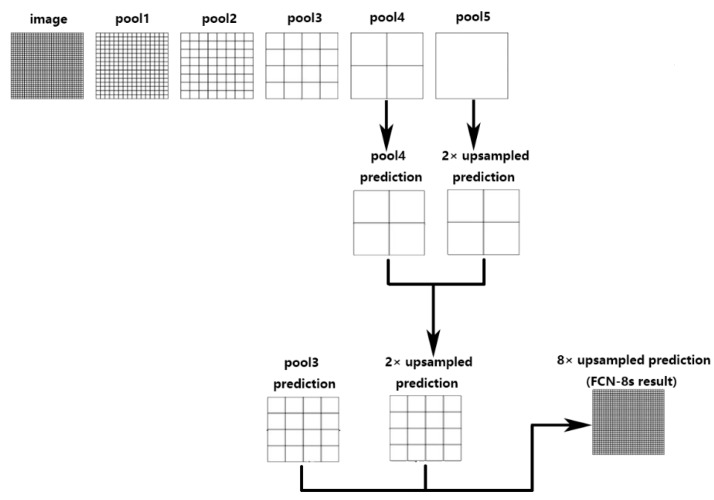
The architecture of FCN-8s.

Figure 2 shows the architecture of FCN-8s, we firstly perform five down-sampling operations, each down-sampling operation consists of two same convolution operations, which have a 3 × 3 kernel size, and one pooling operation. The result is five pooling layers, and each pooling layer is 1/2 the size of the previous pooling layer. Next, the 2× up-sampling feature is obtained by performing up-sampling for the pool5 layer, and then the result is added with the pool4 layer point by point, then up-sampling is performed and added with the pool3 layer point by point. Finally, we get the reconstructed image by 8× up-sampling compared to the pool5 layer.

**Figure 3:**
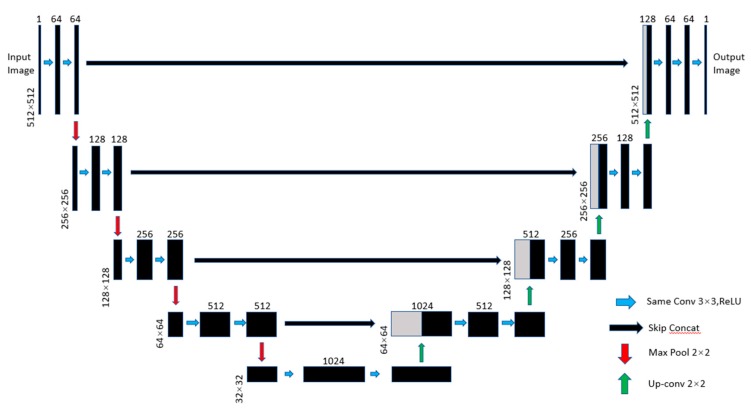
The architecture of U-Net.

Figure 3 shows the architecture of U-Net. The left part of U-Net is a down-sampling process, which is divided into four groups of convolution operations (blue arrow). In each group of down-sampling operation, first performed is the same convolution with a 3 × 3 kernel size, followed by the BatchNormalization operation, and ReLU activation. After each group of convolution operation, there is a max-pooling layer operation (red arrows), the image size will be reduced to 1/2 of the previous step. The input image of 512 × 512 × 1 size was calculated to be 32 × 32 × 1024 size through four groups of operations. The right part shows the up-sampling process. This process included four steps of deconvolution (green arrows). Each deconvolution step expands the image to twice the size of the image in the previous step. Then, the feature image of the corresponding layer is copied and then concatenated to the result of the deconvolution (black arrows).

**Figure 4:**
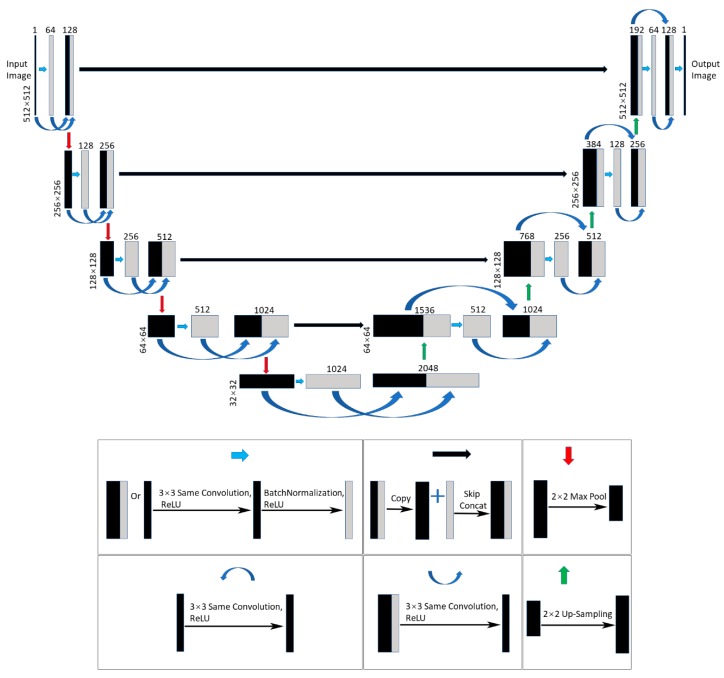
The architecture of Dense-U-Net. Its improvements to U-Net are that there are some densely connected parts in Den-U-Net. The resulting feature layer generated by the same convolution with a 3 × 3 kernel size and the BatchNormalization operation is concatenated with the feature layer generated by the same convolution with a 3 × 3 kernel size.

Figure 4 shows the architecture of Dense-U-Net, the down-sampling and up-sampling processes are the same as those of U-Net. Its improvements to U-Net are that there are some densely connected (curved arrow) parts in the Den-U-Net. The resulting feature layer is generated by the same convolution with a 3 × 3 kernel size and the BatchNormalization operation is concatenated with the feature layer generated by the same convolution with a 3 × 3 kernel size. Then, the feature layer we get is used for the next group of the sampling operation. These densely connected operations enable data of the same size feature layer to be more fully mined, which enables the network to retain more information about the pixel position for the next round of sampling.

## 3. Experimental Results

Our experiment used the following equipments:

Image sensor: We removed the lenses from the original vc-25mc-m30 camera of Viework Company and made a mobile platform to fix it. The processed optical mask was placed 2 mm in front of the CMOS sensor of the camera. The sensor has 5120 × 5120 pixels. The size of the CMOS sensor array is 23.04 mm × 23.04 mm, and each pixel unit size was 4.5 µm × 4.5 µm, with grayscale mode.

Mask material: We used a custom chrome-plated quartz mask, which consists of a fused quartz plate with one side covered with a pattern using a thin chrome film. The pattern of the mask is determined by m-sequence algorithm. The transparent area of the mask transmits light, while the chrome area blocks light. The size of the coded mask is 15.3 mm × 15.3 mm and the size of each aperture is 30 μm × 30 μm.

**Figure 5:**
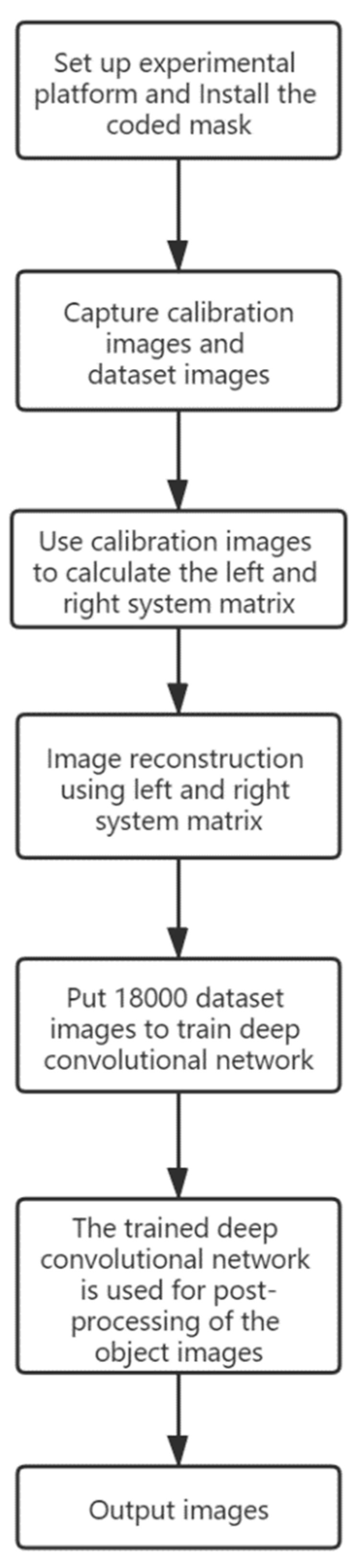
The flow chart of our experimental process.

We trained the deep convolutional network using a machine with a GTX 1060 graphics card and 16 GB of memory. Under these conditions, 18,000 images were fed, and each epoch of training took 40 min for FCN-8s, 1 h and 40 min for U-Net, 2 h and 50 min for Dense-U-Net. All networks have been trained for 6 epochs. In the process of image testing, all the trained networks need about 0.1~0.2 s for post-processing an image. During the acquisition phase of calibration images and data sets, we use a common LCD screen as a light source, the screen brightness was kept at approximately 150 nits and the exposure time was 6 ms. After the network model is trained, passive imaging can be achieved. Figure 5 shows a flow chart of our experimental process.

**Figure 6:**
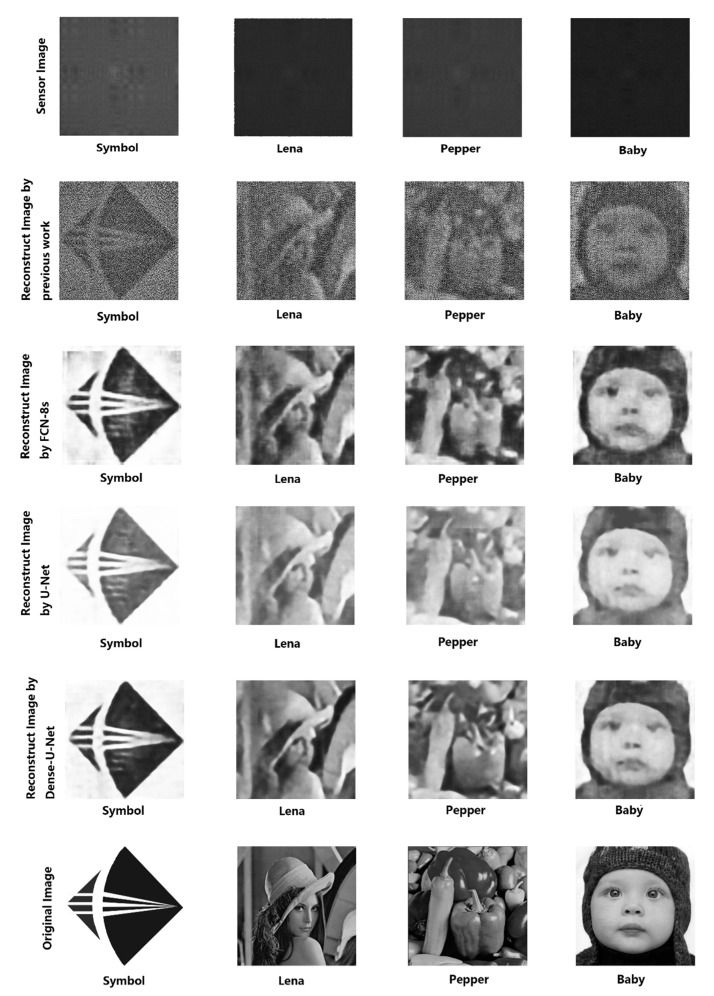
The figure above shows images captured by the sensor, the output results of different methods, and the original scene images. It can be seen that in terms of visual effect, the images processed by FCN-8s network are rough and the details are not recovered accurately, while the grayscale value of the images processed by U-Net network is not very accurate, and the image details and grayscale value processed by Dense-U-Net are relatively accurate.

While our camera has 5120 × 5120 pixels, we firstly cropped and evenly subsampled the sensor images to obtain 512 × 512 images captured by the sensor, then subtracted the mean of the rows and columns from the images. We reconstructed 512 × 512 grayscale images from the 512 × 512 grayscale sensor measurements. The reconstructed results of the calibration images are used to calculate the left and right system transfer matrix described in 2.1, which we use to reconstruct the object image $\hat{X}$. Since recent years, the deep learning method has many effective applications in the field of reconstructing degraded images caused by various reasons, we regarded $\hat{X}$ as a degraded image, and put it into deep convolutional networks for post-processing. Figure 6 shows some sensor images and the reconstruction results of previous work, FCN-8s, U-Net, and Dense-U-Net. Table 1 shows four image evaluation parameters, PSNR (Peak Signal to Noise Ratio), SSIM (Structural Similarity Index), NQM (Noise Quality Measure), and FSIM (Feature Similarity Index) of output images produced by different reconstruction processes and networks. We can see that the image quality of output images post-processed by the deep convolutional network is much better than the image quality of images reconstructed by previous work. The images processed by U-Net performed better than FCN-8s in the image evaluation parameters related to the structure and the noise performance, and worse than FCN-8s in the image evaluation parameters related to the gray value. The overall performance of the image processed with Dense-U-Net is the most outstanding. According to the comparison results, the reconstruction of lensless sensing images by our method can achieve better results in evaluation parameters such as PSNR, SSIM, NQM, and FSIM, and the visual effect of our reconstruction is closer to original scene images than the previous work.

## 4. Discussion

At present, there is still a long gap between the quality of lensless reconstructed images and the quality of images acquired by lens imaging systems. We can only improve the quality of lensless images through a lot of exploration. Obviously, compared with the previous work, the visual effect of our reconstructed images is closer to the original scene, and the results of image evaluation parameters such as PSNR, SSIM, NQM, and FSIM have been greatly improved. The results of our experiment further increase the possibility of replacing the lens camera with a lensless coded mask camera. However, there is still an important problem: The details of the final image are still obscure due to the non-optimal size of the prototype template and the ill-conditioned problem of the system transfer matrix calculation. In order to improve image quality and image resolution, physical imaging factors need to be further analyzed. Another important reason is the inherent insensitivity to details of FCN networks. We can see clearly that the processing of the feature layer by 8× up-sampling directly in the FCN-8s network is not as good as that of U-Net or Dense-U-Net, which conducts 2× up-sampling three times. The reason why the latter two networks can restore more details is their smoother up-sampling steps and skip concatenation steps. These operations let the network make better use of the structural information contained in the previous feature layer. What‘s more, denser concatenating steps allow more contextual information to be disseminated by letting data of the same sized feature layer to be more fully mined and enable the network to retain more information about the pixel position for the next group of sampling. We believe that these properties contribute to improving network performance. Comparing final reconstructed results in the second rows to others in Figure 6, we can see that the method combined with the deep convolutional network can greatly improve the quality of lensless imaging results. Comparing final reconstructed results in the third, fourth, and fifth rows of Figure 6, we can see that images processed by the Dense-U-Net network have smoother edges, more details, and higher image evaluation parameters. Of course, the details of images processed by Dense-U-Net are still not precise enough. It suggests that no matter how many iterative calculations, the down-sampling operations will damage images’ detail, and the up-sampling step cannot restore all pixel’s position information, but the stepwise up-sampling operation can lose as little detail as possible to preserve more pixel’s position information.

## 5. Conclusions

In this paper, we used the deep convolutional networks to post-process images captured by the lensless device and reconstructed by the inverse matrix optimization method, and successfully proved the great potential and value of combining the deep learning with the traditional lensless technology. In addition, we proposed Dense-U-Net by combining the end-to-end U-Net with the denser link thought contained in DenseNet, which further improved the visual effect and image evaluation parameters of the output image. However, there is still a long journey ahead regarding how to improve the quality and resolution and provide more details in lensless images, which are crucial to put this technology into practical use and adopt it in the large-scale Internet of Things and space-constrained scenarios. In the future, we hope to improve the spatial resolution of lensless imaging technology so that more details of the scene can be recovered. We believe the goal is achievable with an increased overall numerical aperture of the mask, and more effective optimization reconstruction algorithms. With the problems of integration being solved and lensless imaging resolution being improved, we can expect a broad application in the field of biological imaging.

## Figures and Tables

**Table 1:** Imaging quality evaluation parameters of output images produced by several methods. The best results are shown in bold.

	PSNR				SSIM			
	Previous work	FCN-8s	U-Net	Dense-U-Net	Previous work	FCN-8s	U-Net	Dense-U-Net
Symbol	6.9629	16.8320	17.7754	**18.7957**	0.0128	0.7872	0.8312	**0.8445**
Lena	10.0581	19.2174	18.9286	**19.8937**	0.0129	0.5547	0.5971	**0.6013**
Pepper	9.8043	18.3765	17.4211	**19.0304**	0.0142	0.5586	0.5733	**0.5805**
Baby	809028	18.3188	17.4833	**18.4121**	0.0133	0.5801	**0.5985**	0.5973

	**NQM**				**FSIM**			
	Previous work	FCN-8s	U-Net	Dense-U-Net	Previous work	FCN-8s	U-Net	Dense-U-Net
Symbol	3.5432	9.0763	10.2099	**11.1406**	0.1971	0.6955	0.7501	**0.7634**
Lena	3.6515	5.8475	6.6642	**7.0634**	0.4822	0.7279	0.7391	**0.7423**
Pepper	3.5218	6.2367	7.3112	**7.9167**	0.4828	0.7384	**0.7568**	0.7548
Baby	3.7463	7.670	7.7607	**8.0797**	0.4943	0.7531	0.7686	**0.7595**
